# Influence of Periodontal Ligament Stem Cell-Derived Conditioned Medium on Osteoblasts

**DOI:** 10.3390/pharmaceutics14040729

**Published:** 2022-03-28

**Authors:** Solen Novello, Sylvie Tricot-Doleux, Agnès Novella, Pascal Pellen-Mussi, Sylvie Jeanne

**Affiliations:** 1 ISCR (Institut des Sciences Chimiques de Rennes)—UMR 6226, Université de Rennes, 35000 Rennes, France; sylvie.tricot@univ-rennes1.fr (S.T.-D.); agnes.novella@univ-rennes1.fr (A.N.); pascal.pellen@univ-rennes1.fr (P.P.-M.); sylvie.jeanne@univ-rennes1.fr (S.J.); 2 Unité de Formation et de Recherche d’Odontologie, Université de Rennes, 35000 Rennes, France; 3 UF Parodontologie, Pôle d’Odontologie, Centre Hospitalier Universitaire de Rennes, 35000 Rennes, France

**Keywords:** mesenchymal stem cells, conditioned medium, exosomes, osteoblasts, periodontal regeneration, bone regeneration

## Abstract

Mesenchymal stem cells (MSC) are involved in the regeneration of various missing or compromised periodontal tissues, including bone. MSC-derived conditioned medium (CM) has recently been explored as a favorable surrogate for stem cell therapy, as it is capable of producing comparable therapeutic effects. This study aimed to evaluate the influence of periodontal ligament stem cells (PDLSC)-CM on osteoblasts (OB) and its potential as a therapeutic tool for periodontal regeneration. Human PDLSC were isolated and characterized, and CM from these cells was collected. The presence of exosomes in the culture supernatant was observed by immunofluorescence and by transmission electron microscopy. CM was added to a cultured osteoblastic cell line (Saos-2 cells) and viability (MTT assay) and gene expression analysis (real-time PCR) were examined. A cell line derived from the periodontal ligament and showing all the characteristics of MSC was successfully isolated and characterized. The addition of PDLSC-CM to Saos-2 cells led to an enhancement of their proliferation and an increased expression of some osteoblastic differentiation markers, but this differentiation was not complete. Saos-2 cells were involved in the initial inflammation process by releasing IL-6 and activating COX2. The effects of PDLSC-CM on Saos-2 appear to arise from a cumulative effect of different effective components rather than a few factors present at high levels.

## 1. Introduction

Periodontitis is a bacterially-induced chronic inflammatory disease of the periodontium [1]. The accumulation of dental plaque microorganisms can result in the progressive and irreversible destruction of tooth-supporting tissues, including gingiva, periodontal ligament, cementum, and alveolar bone. This disease causes loss of bone and soft tissue attachment and can lead to dental mobility. If left untreated, periodontitis can result in premature tooth loss [2]. Regeneration of periodontal tissues is the ultimate goal of periodontal treatment [1]. Conventional nonsurgical or surgical treatment can arrest and treat the periodontal disease [3]. However, complete and functional periodontal regeneration cannot be fully achieved with current therapeutic approaches and is still a clinical challenge [4].

In recent years, extensive research has been carried out on the therapeutic efficacy of mesenchymal stem cells (MSC). They have been shown to play a major role in bone repair, thanks to their unique capabilities of self-renewal and differentiation. They therefore appear to be a promising candidate for bone tissue engineering. MSC can be isolated from a wide range of tissues. Bone marrow and adipose MSC were first widely explored, but soon technical limitations led the research teams to seek an alternative source of MSC, among them dental tissues [5]. Dental MSC have several advantages over other stem cells: easy isolation by noninvasive routine clinical procedures (e.g., extraction of third molars) and so a limited morbidity; a higher proliferation rate; an increased survival in culture with a delayed senescence process compared to bone marrow MSC and the absence, to date, of major adverse reactions [6,7]. The use of dental MSC has shown therapeutic benefits in many medical fields: amelioration of ischemic tissue injury and acceleration of functional recovery after ischemic stroke [8], promotion of angiogenesis and vasculogenesis [9], prevention of the progression of liver fibrosis and contribution of the restoration of liver function [10], or even reconstruction of the corneal epithelium [11]. Transplantation of periodontal ligament stem cells (PDLSC) has enhanced periodontal regeneration in animal models [12,13], and human clinical trials are currently underway. The first results show that there may be a positive impact of MSC-based therapy on periodontal regeneration [3].

However, the literature reveals that the effects of MSC in tissue engineering is largely related to a paracrine action by the secretion of various factors, such as growth factors, cytokines, chemokines, enzymes, or extracellular vesicles that include exosomes [14,15,16]. Moreover, transplanted MSC do not survive for a long time, and the engraftment is limited. Since then, a growing number of studies have focused on the conditioned medium (CM) of mesenchymal stem cells and its therapeutic potential [17,18,19,20]. The therapeutic outcomes of MSC could be duplicated by MSC-CM that contains the secretome of the cells, while circumventing the limitations associated with stem cell therapy [5,21,22].

Based on this information, we hypothesized that transplantation of paracrine factors from PDLSC could induce bone regeneration and play a role in the regeneration of periodontal tissues. This can involve MSC that differentiate into osteoblasts (OB) or primary OB. We decided to study the effects and interactions of PDLSC-CM on an osteoblastic cell line: Saos-2 cells. These cells possess a high biomineralization capacity, and their ability to deposit a mineralization-competent extracellular matrix makes them as a valuable candidate for studying the stage of differentiation into OB [23]. Markers of osteogenesis, angiogenesis and inflammation, key events to address during periodontal regeneration, were investigated, as well as heat shock proteins (HSP), proteins synthesized in response to a variety of stresses, such as inflammation, microbial infection, diseases, or variations in temperature or pH [24,25]. It has been shown, for example, that the temperature in inflamed periodontal pockets is higher than that in healthy sites [26]. It is well known that pro-inflammatory cytokines, such as IL-1, TNF-α and INF-γ, are produced in periodontitis and these cytokines could cause an elevation of the levels of HSP in the inflamed periodontium [24,25]. HSP are grouped into families according to their molecular weight: HSP60 (60-kDa HSP), HSP70, HSP90, etc., and act as molecular chaperones to protect cells from abnormal conditions [25,26]. They exert potent intercellular signaling activities with properties similar to those of both pro- and anti-inflammatory cytokines, and they have recently been proposed as useful biomarkers [27].

This study aimed to evaluate the influence of PDLSC-CM on Saos-2 cells and its potential as a therapeutic tool for periodontal regeneration.

## 2. Materials and Methods

Three independent experiments were performed for each condition.

### 2.1. Human Periodontal Ligament Stem Cells

#### 2.1.1. Isolation and Culture

Human Periodontal Ligament Stem Cells (hPDLSC) were obtained using the explant outgrowth method. Impacted third molars were collected from a healthy donor in agreement with French legislation (informed patients and Institutional Review Board approval/Registration number: DC-2012-1573) and stored at 4 °C in Dulbecco’s modified Eagle’s medium (DMEM, Lonza, Verviers, Belgium) supplemented with antibiotics (100 U/mL penicillin, 100 μg/mL streptomycin, Lonza, Verviers, Belgium), antifungal (5 μg/mL amphotericin B, Lonza, Verviers, Belgium), and 20 mmol/L HEPES (Lonza, Verviers, Belgium). The periodontal ligament was removed from the teeth aseptically, rinsed with DMEM, and explanted in a 35-mm Petri dish at 7–8 small pieces/well (≈1 mm^3^). Explants were grown in a complete culture medium composed of DMEM supplemented with 10% fetal bovine serum (FBS, Gibco, Life Technologies, Paisley, UK), 2 mmol/L glutamine (Lonza, Verviers, Belgium), 20 mmol/L HEPES, and antibiotics (100 U/mL penicillin, 100 μg/mL streptomycin) until human periodontal ligament cells emerged from the explant. These cultures were maintained at 37 °C in a humidified atmosphere of 5% CO2. Cells were used from passage 3 to 7.

#### 2.1.2. Characterization

##### Phenotypic Characterization by Flow Cytometry

Flow cytometric analysis was performed for immunophenotypic characterization. After washing with phosphate buffer saline (PBS) (Lonza, Verviers, Belgium), the cell pellet was centrifuged for 5 min at 300 g. The cells were incubated with BV421 mouse anti-human CD34, FITC mouse anti-human CD45, PE mouse anti-human CD73, PE-Cy7 mouse anti-human CD90, and APC mouse anti-human CD105 for 15 min at room temperature (RT). After incubation, the cells were washed with PBS for further analysis. The expression profiles were examined using a BD LSRFortessa™ X-20 flow cytometer system (BD Biosciences, San Jose, CA, USA). Antibodies and buffers were purchased from BD Biosciences (San Jose, CA, USA).

##### Functional Characterization

Colony-Forming Assay 

Cells were seeded at a density of 500 cells/well in 6-well plates and cultured in complete medium. After 10 days, they were stained with 0.5% crystal violet in methanol 100% for 5 min and washed with distilled water [28]. The colonies were then observed.

Osteogenic Induction

To assess OB differentiation potential, cells were seeded at a concentration of 0.5 × 10^5^ cells/mL in 6-well plates in complete medium. After 24 h, the medium was switched to an osteoinductive medium consisting of complete medium supplemented with 50 μg/mL of ascorbic acid (Sigma-Aldrich A4403, Saint-Louis, MO, USA), 10 mM of β-glycerophosphate (Sigma-Aldrich G9422, Saint-Louis, MO, USA), and 100 nM of dexamethasone (Sigma-Aldrich D4902, Saint-Louis, MO, USA). The medium was changed every 2 or 3 days. After 21 days, cells were stained with 40 mM Alizarin Red solution (Sigma-Aldrich A5533, Saint-Louis, MO, USA) for 20 min at RT and washed with distilled water.

Adipogenic Induction

For adipogenesis experiments, cells were seeded at a concentration of 0.5 × 10^5^ cells/mL in 6-well plates in complete medium. After 24 h, the medium was switched to an adipocyte differentiation medium consisting of complete medium supplemented with 100 μM indomethacin (Sigma-Aldrich I7378, Saint-Louis, MO, USA), 0.5 mM 3-isobutyl-1-methylxanthine (Sigma-Aldrich I5879, Saint-Louis, MO, USA), 1μM dexamethasone (Sigma-Aldrich D4902, Saint-Louis, MO, USA), and 1 μM insulin (Sigma-Aldrich I1882, Saint-Louis, MO, USA). Medium was changed every 2 or 3 days. After 21 days, cells were stained with Oil Red O solution diluted at 60% in distilled water (Sigma-Aldrich O1391, Saint-Louis, MO, USA) for 30 min at RT and washed with distilled water.

#### 2.1.3. Exosomes

##### Isolation

hPDLSC were cultured in 75 cm^2^ flasks in complete DMEM. When reaching 70% confluency, the culture medium was switched to serum-free DMEM. Cell culture supernatants were collected at day 3. The exosome isolation kit ExoQuick-TC was used according to the manufacturer’s instructions (System Biosciences, Palo Alto, CA, USA). In brief, an initial spin was performed at 3000× *g* (RT) for 15 min for each sample to remove cells and debris, then the corresponding amounts of reagents were added proportional to the starting sample volume. Mixtures were agitated and incubated at 4 °C overnight. They were then centrifuged at 1500× *g* (RT) for 30 min to precipitate exosome pellets, followed by pellet resuspension in PBS. The resuspension volume for exosome pellets was 150 μL for 10 mL starting volume. All exosomes were stored at −80 °C immediately after isolation until further analysis. For TEM, samples were freshly isolated for better image quality.

##### Fluorescence Immunodetection

Cells were plated in a Labtek Chamber slide at a density of 2 × 10^4^ cells/mL After three days, when the cells had reached near confluency, medium was removed from the cells and they were washed with PBS. After fixation with methanol 100% for 5 min, hPDLSC were incubated over night at 4 °C with primary antibodies. The antibodies used in this study include the following: anti-CD9 (monoclonal mouse, IgG1k, 1:1000, BioLegend, San Diego, CA, USA), anti-CD63 (monoclonal mouse, IgG1k, 1:500, Santa Cruz Biotechnology, Inc., Dallas, TX, USA), and anti-ALIX (monoclonal mouse, IgG1k,1:500, Santa Cruz Biotechnology, Inc., Dallas, TX, USA). For negative controls, the primary antibody was replaced by non-immune serum (mouse IgG, 1:500, Sigma-Aldrich, Saint-Louis, MO, USA). Then a secondary goat anti mouse AlexaFluor488 antibody (LSBio, Seattle, WA, USA) was applied for 1 h at RT. Counterstaining was performed using Hoechst-dye (1:2000, Sigma-Aldrich, Saint-Louis, MO, USA). Observations were performed using an Upright Widefield Microscope Leica DMRXA2 (Leica Microsystemes, Wetzlar, Germany).

##### Transmission Electron Microscopy

For transmission electron microscopy, freshly isolated exosome suspensions were fixed in 4% paraformaldehyde. An amount of 10 μL of exosome suspensions were applied onto a carbon-Formvar coated grid for 20 min at RT, then rinsed in Tris-HCl buffer (TBS, Sigma-Aldrich T5912, Saint-Louis, MO, USA). To observe exosome morphology, as a final step, the samples were negatively stained with 4% uranyl acetate for 10 min, and grids were wicked dry and then allowed to air dry. For immunoelectron microscopy, samples were incubated with blocking buffer (5% TBS in BSA) for 10 min. Grids were then incubated with either blocking buffer only (negative control) or primary antibody (anti-CD9, anti-CD63, or anti-ALIX) diluted with blocking buffer for 45 min. After rinsing in TBS/BSA, the samples were incubated with secondary antibody conjugated with 10-nm Protein A gold (1/20) for 45 min. After rinsing in TBS, the samples were negatively stained, as already described. Sample TEM examination was performed using a JEOL 1400 TEM (JEOL, Tokyo, Japan).

### 2.2. Conditioned Medium

#### 2.2.1. Preparation

Monolayers hPDLSC were seeded at a density of 1.25 × 10^4^ cells/mL into 6-well plates. They were cultured in 10 mL of complete DMEM without FBS. Culture supernatants were collected after 48 h of incubation and centrifuged at 2000× rpm for 5 min. After being collected and filtered in 0.22 µm filters, CM was concentrated 100-fold using ultrafiltration with a 3 kDa cut-off value (AMICON Ultra-15 3K, Merck Millipore, Darmstadt, Germany), at 4000× *g* during 60 min at RT; hPDLSC-CM was then divided in aliquots that were stored at −80 °C until use [29,30,31]. As a control, cell-free medium was prepared in the same way (control-CM).

#### 2.2.2. Protein Concentration

The protein concentration in hPDLSC-CM was measured using a Micro BCA Protein Assay Kit (Thermo Scientific Pierce, Thermo Fisher Scientific, Rockford, IL, USA), according to the manufacturer’s instructions. The absorbance at 570 nm was read with a spectrophotometer (Sunrise, Tecan, Männedorf, Switzerland). 

#### 2.2.3. Enzyme-Linked Immunosorbent Assay

Concentrations of IL-6 and IL-8 in the CM collected from hPDLSC were investigated using enzyme-linked immunosorbent assay (DuoSet ELISA Development System Human IL-6 and IL-8, R&D Systems, Minneapolis, MN, USA) according to the manufacturer’s instructions. The absorbance at 450 nm was read with a spectrophotometer. Concentrations were calculated from the standard curves. Assay range was 9.4 to 600 pg/mL for IL-6 and 31.2 to 2000 pg/mL for IL-8. As a control, the levels of IL-6 and IL-8 in medium with 10% FBS or without FBS, in control-CM and in culture supernatants before concentration, were measured.

### 2.3. Culture of Saos-2 Cells

Saos-2 cells, a human osteoblastic cell line, were obtained from ATCC (Manassas, VA, USA). They were amplified in complete DMEM. The cells were cultured in a humidified atmosphere containing 5% CO_2_ at a temperature of 37 °C. 

#### 2.3.1. MTT Assay

The relative growth of Saos-2 cells was evaluated using the MTT assay, as previously described [32]. Briefly, Saos-2 cells were seeded in 96-well tissue culture plates in 200 µL medium/well, at a concentration of 3.5 × 10^4^ cells/mL. After 24 h, the cells were incubated for 48 or 72 h with different concentrations of hPDLSC-CM diluted in complete medium without FBS: 62.5 µg/mL, 125 µg/mL, 250 µg/mL, or 500 µg/mL. Saos-2 cells were then exposed to MTT (1 mg/mL) for 3 h at 37 °C. After complete solubilization of formazan crystals in Dimethyl sulfoxide, optical density was measured on an enzyme-linked immunosorbent assay plate reader at 570 nm. Control cells were cultured in medium containing 10% or 0% FBS.

#### 2.3.2. RNA Extraction and Real-Time Polymerase Chain Reaction (RT-qPCR)

Saos-2 cells were seeded at a density of 5 × 10^4^ cells/mL into 6-well plates. After 24 h, the medium was changed and cells were incubated in complete DMEM without FBS; hPDLSC-CM (250 µg/mL) was added to the medium and the cells were cultured for 72 h.

Total RNA was extracted from Saos-2 cells by the Ribozol™ RNA extraction reagent (AMRESCO, Solon, OH, USA). The RNA concentration was quantified by absorbance at 260 nm and checked by optical density ratio at 260/280 nm (1.8 < ratio < 2) and 260/230 nm (2 < ratio < 2.2). Total RNA was reverse transcribed into complementary deoxyribonucleic acid (cDNA) using the cDNA synthesis kit Protoscript First Strand cDNA Synthesis Kit^®^ (Biolabs E6560, New England Biolabs, Ipswich, MA, USA). Quantitative RT-PCR was carried out with a SYBR^®^ Green PCR kit (Applied Biosystems, Foster City, CA, USA) in an a QuantStudioTM 7 Pro system (Applied Biosystems, Life Technologies LTD, Singapore) under the following cycling conditions: 2 min at 50 °C; 10 min at 95 °C; 40 cycles of 15 s at 95 °C and 1 min at 60 °C; and a final dissociation step. The primer sequences used in this experiment are listed in Table 1. The transcripts of the *18S* and *HPRT* housekeeping gene were used to normalize the mRNA levels [33,34]. The results were normalized using the geometric mean Ct of *HPRT* and *18S*. Quantitative results were analyzed with Design and Analysis software v2.6.0 (Applied Biosystems, Life Technologies LTD, Singapore). The assay for each gene was carried out in triplicate. The validation of the primers, the purity and integrity of the RNA, and the amplification efficiency have been verified according to the recommendations of Taylor and Mrkusich [35].

### 2.4. Statistical Analysis

Data analysis was performed with GraphPad Prism v9.3.1 (GraphPad Software, San Diego, CA, USA). One-way or two-way analysis of variance and Tukey’s post hoc tests were used to determine significant differences between groups. Data are expressed as the means ± standard deviation, and differences were considered significant when *p* < 0.05.

## 3. Results

### 3.1. Isolation and Characterization of hPDLSC

Prior to the experiments, it had been confirmed that hPDLSC, isolated with the explant outgrowth method, showed MSC-like characteristics. They exhibited a spindle appearance, similar to those of MSC.

Cytometric flow analysis revealed positive expression of CD73, CD90 and CD105, and negative expression of CD34 and CD45 (Table 2).

**Figure 1:**
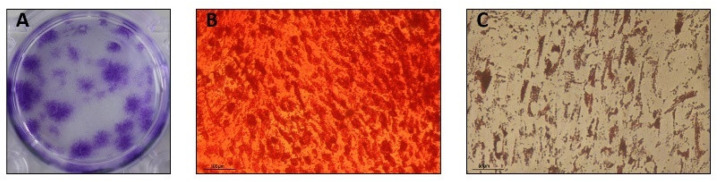
Characterization of hPDLSC. (**A**) Colony-forming assay. (**B**) Alizarin red staining (Scale bar = 100 µm). (**C**) Oil Red O staining (Scale bar = 50 µm).

The hPDLSC were able to form colonies ten days after seeding (Figure 1A). Additionally, the osteoblastic differentiation potential was confirmed. These colony-forming cells produce nodules of mineralization revealed by Alizarin Red staining after 21 days (Figure 1B). Similarly, in the presence of adipocyte differentiation medium, we could observe the formation of lipid droplets, revealed by Oil Red O staining (Figure 1C).

### 3.2. Isolation and Characterization of hPDLSC-Derived Exosomes

**Figure 2:**
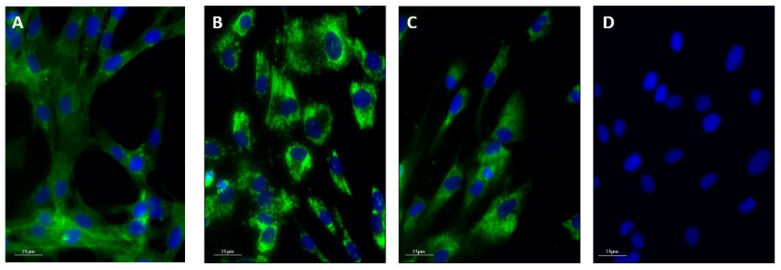
Immunofluorescence staining of hPDLSC with CD9 (**A**), CD63 (**B**), and ALIX (**C**). Negative control with non-immune serum (**D**). Objective 40×/1.0 PL Fluotar. Scale bars = 25 µm.

In order to confirm the presence of exosomes in the culture supernatant, we looked for the expression of appropriate markers in hPDLSC by immunocytochemical analysis. Expression of all three markers were observed showing a spot-like pattern in the cytoplasm (Figure 2).

**Figure 3:**
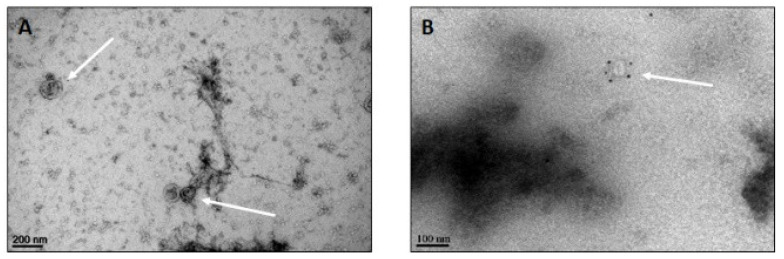
TEM observation: exosomes (**A**) and example of immunogold labeled exosomes with anti-CD63 (**B**). Scale bars = 200 nm (**A**) and 100 nm (**B**).

Ultrastructural investigation of isolated exosomes revealed the expected size distribution and membrane integrity. Some exosomes are spherical; others are heterogeneous in shape (Figure 3A). The appearance of relevant exosomal markers were visualized by immunogold labeling. Black punctate regions indicate a positive staining of CD9, CD63 and ALIX (Figure 3B).

### 3.3. Conditioned Medium

**Figure 4:**
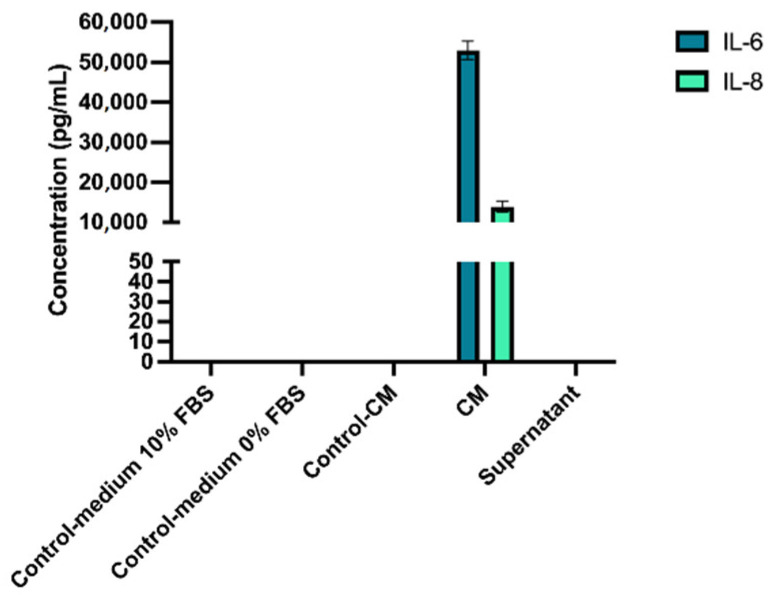
Expression of IL-6 and IL-8 in hPDLSC-CM, compared with supernatant and cell-free medium.

To evaluate the effectiveness of our concentration technique, levels of IL-6 and IL-8 in hPDLSC-CM were measured and compared with levels in culture supernatants. The concentration of IL-6 was 52,980.5 ± 2279 pg/mL and IL-8 was 13,923.2 ± 1403.7 pg/mL (mean ± sd) (Figure 4). Quantities were not detectable in control medium, control-CM, or supernatant because they were lower than the minimal amount detectable with the kit used (<9.4 pg/mL for IL-6 and <31.2 pg/mL for IL-8).

The average protein concentration in hPDLSC-CM was 3762 ± 111.7 µg/mL (mean ± sd).

### 3.4. Influence of hPDLSC-CM on SaOS-2 Cells

#### 3.4.1. Optimal Time and Concentration of CM

**Figure 5:**
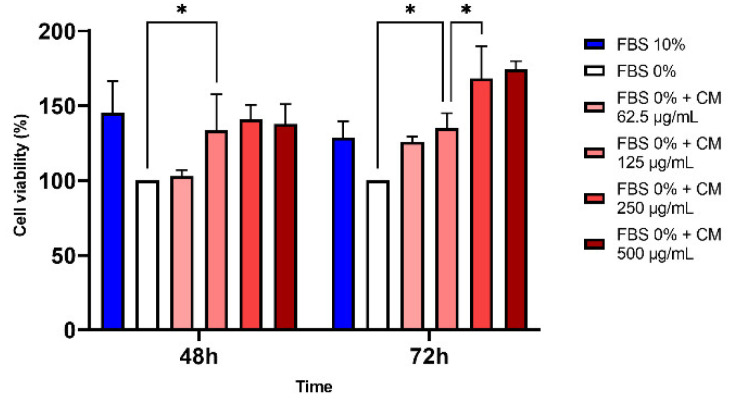
Cell viability of Saos-2 analyzed by MTT assay after 48 or 72 h of treatment with different concentrations of CM (* *p* < 0.05).

The addition of hPDLSC-CM increased Saos-2 cell proliferation. At 48 h, we noticed a significant difference with control cells cultured in medium without FBS from a concentration of 125 µg/mL, but no difference with higher concentrations. At 72 h, cell proliferation was increased from a concentration of 62.5 µg/mL and rose with higher concentrations, exhibiting a dose-response effect. The optimal growth was observed at 72 h, with a concentration of 250 µg/mL or 500 µg/mL. Because no difference was found between the two, the parameters chosen for the rest of the experiments were 72 h and 250 µg/mL (Figure 5).

#### 3.4.2. Saos-2 Gene Expression

**Figure 6:**
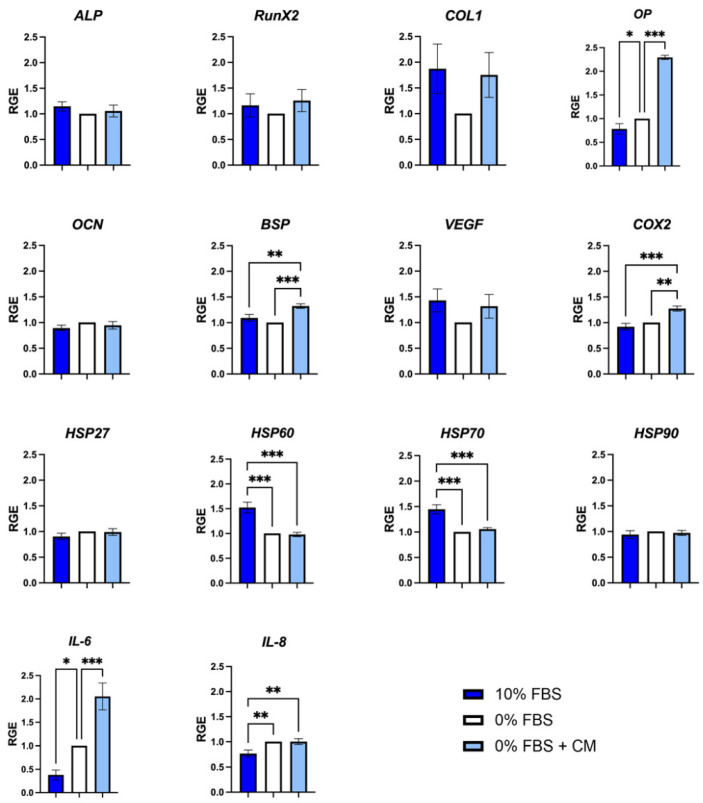
Quantitative reverse transcription-polymerase chain reaction demonstrating the effect of hPDLSC-CM on Saos-2 gene expression. RGE: Relative Gene Expression. * *p* < 0.05; ** *p* < 0.01; *** *p* < 0.001.

Markers of osteogenesis, cell stress, and inflammation were explored by quantitative RT-qPCR (Figure 6). The markers investigated can be classified into three categories:

- Those for which the expression did not vary according to the conditions: ALP, OCN, HSP27 and HSP90;
- Those for which FBS deprivation had an impact: *COL1*, *HSP60*, *HSP70* (decrease of the expression), *IL-6* and *IL-*8 (increase of the expression);
- Those for which the addition of CM increased expression compared to the control medium (FBS 0%): *COL1*, *OP*, *RunX2*, *BSP*, *VEGF*, *COX2* and *IL-6*.

Compared to the serum-free medium, the addition of CM gave either no effect or an increase in expression. No gene expression variation was detected between cells cultured with serum-free DMEM and serum-free DMEM supplemented with control-CM (data not shown).

## 4. Discussion

In this study, a cell line with all the characteristics of MSC was isolated and characterized. Phenotypic (flow cytometry) and functional (CFU assay and differentiation potential) characterization has allowed objectifying the presence of MSC in periodontal ligament [36].

The use of MCS secretome appears to be an alternative to cell-based therapy for bone and periodontal tissue regeneration [37]. In order to evaluate the influence of paracrine factors from hPDLSC on bone regeneration, we decided to use human osteoblastic Saos-2 cells. Despite their ineligibility for clinical use owing to their tumor derivation, Saos-2 cells were chosen for this study as they are easy to obtain and handle and are a widely diffused and accepted in vitro model in the field of bone biology [38,39]. They allow studying the OB differentiation process because they have a high biomineralization capacity and they produce an extracellular matrix compatible with mineralization [23]. They show responses resembling those of primary human OB and immortalized osteoblastic cell lines (such as hFob 1.19 transfected with a temperature-sensitive SV40 T-antigen) with respect to osteoblastic marker expression, while avoiding a possible temperature effect on the differentiation [38,40]. This line has a cell cycle and a regular proliferation, allowing it to have a reproducible effect, which is not the case with primary OB that present inter-individual variations depending mainly on the age of the patient.

We opted for the use of CM instead of purified exosomes, containing the secretome of the cells: extracellular vesicles, but also growth factors, cytokines, and other active substances. hPDLSC were cultured in serum-free medium, FBS containing a large number of exosomes [41]. The presence of exosomes in the culture supernatant was objectified by immunofluorescence observed with confocal microscopy and by TEM structural observation and immunodetection of relevant exosomal markers [42,43,44]. Our method to obtain CM has been validated by comparing the concentration of two markers, IL-6 and IL-8, by ELISA. A growing number of research teams are working on the therapeutic potential of CM, in many medical fields [5,45,46]. MSC-CM transplantation, as a cell-free technique, is ready-to-use and more convenient than using exosomes [30]. Currently, there is no optimal purification technique for the isolation of exosomes with high purity. Most isolation methods yield only a small amount, making it more complicated for clinical translation [37]. Moreover, the purification kit we used in this study (ExoQuick-TC) is not recommended for in vivo use [47].

The control used for gene expression analysis (CM without cells) confirmed that the observed effect was due to the cellular secretion of different elements in the supernatant and that the concentration method does not have a biological effect. We performed some tests with control-CM, showing no difference with cells grown in serum-free medium.

According to the literature, we chose a contact time of 72 h maximum [1,48]. Depending on the studies, RNA extractions are most often carried out after 48 or 72 h of CM contact [49,50,51].

Our results showed a positive effect of CM on the proliferation of Saos-2, with an increase of more than 70%. This agrees with Park et al., who showed a dose-dependent effect of CM [52]. However, the results of Aghamohamadi et al., who investigated the effect of different concentration of PDLSC-CM on the proliferation of PDLSC, showed that high concentrations may cause a significant decrease in cell proliferation [48]. This is consistent with the study of Paschalidis et al., who evaluated two different dilutions (50% and 100%) of MSC-CM on dental pulp stem cells proliferation and reported a greater result for the 50% dilution [53]. In our study, we observed a plateau effect above 250 µg/mL. By increasing the concentrations, we might have observed a decrease. It is therefore essential to define the optimal concentration of CM to obtain a balance between the positive effect of paracrine factors and the possible metabolic inhibitory effect of by-products [48].

Both MSC and OB are essential for the promotion of osteogenesis. Through the secretion of numerous mediators, MSC interact with OB and can interfere with their differentiation. Depending on the study, the observed effects seem contradictory. Some observed that MSC repress OB differentiation [54,55,56]. The results of Santos et al. showed that bone marrow MSC-CM (BMMSC-CM) reduce OB proliferation, downregulate bone marker genes, and inhibit mineralized matrix formation [54]. Sun et al. demonstrated that BMMSC may suppress OB proliferation and transiently retard OB differentiation [55]. Conversely, other studies have shown that MSC secretome promote bone regeneration [49,51,57,58,59]. The effects, though, are more obvious when CM or extracellular vesicles come from MSC cultured in osteogenic medium [60,61]. Yahao et al. compared the effect of exosomes derived from standardized MSC culture and from osteogenic induction medium on OB and observed that the second ones had more obvious effect. They showed that exosomes are influenced by the differentiation stage of the cell of origin and carry related substances to promote osteoblast differentiation [61]. However, these latest studies evaluate the influence of MSC on the osteogenic differentiation of undifferentiated MSC and not on OB, which may explain the differences observed in results. In our study, the expression of osteogenesis markers is increased under the influence of CM, except for *ALP* and *OCN*, where no effect is observed. For *RunX2* and *COL1*, this increase is not significant. It is, however, for *OP* and *BSP*. These results indicate that osteogenesis is not complete at 72 h. Lan et al. demonstrated that the increase in *ALP* and *OCN* expression occurred only after 6 days of contact with a medium containing exosomes [62]. In their study, Jin et al. observed an increase in *ALP* expression after 10 days of coculture with MSC-CM [60].

Angiogenesis contributes to the progression of osteogenesis, where blood supply induces OB migration and mineralization of bone tissue. Our results showed that the addition of CM increased, though not significantly, the expression of *VEGF*, which is a specific growth factor that promotes proliferation and migration of vascular endothelial cells, revascularization and capillary production, and improves the cellular activity of osteoblasts, thereby promoting bone regeneration [49].

The first objective of the periodontal treatment is to control the inflammation in order to promote high-quality healing. It is suggested that exosomes or CM from MSC exerts anti-inflammatory effects during periodontal wound healing [29,30,63].

The initial increase in *COX2* and *IL-6* expression shows the need for an inflammatory response in the initial stages of osteogenesis and tissue repair. This phenomenon allows, in particular, attraction by chemotaxis of the debridement cells (professional phagocytes) and the cells of repair (bone progenitor cells or stem cells). In their study, Yoon et al., showed that *COX2* inhibitors suppress bone repair and bone formation [64].

A study by Al-Sharabi et al. showed that MSC-CM promote the secretion of inflammatory molecules in vitro and, conversely, attenuates the initial inflammatory response in vivo [65]. These authors demonstrated an important increase of IL-6 and IL-8 in the supernatant from CM-treated dental pulp cells, as in our study, thanks to a Multiplex assay in vitro. Al-Sharabi et al. explain that this paradox between cell cultures and in vivo experiments can be attributed to the dominant inflammation-regulating effect of IL-10 in vivo, which increases over time. IL-10 is a key inflammatory cytokine, having an immunoregulatory effect. It regulates the secretion of pro-inflammatory cytokines and plays an important role in suppressing inflammatory and immune responses. This is confirmed in the study of Qiu et al., who highlight the impact of MSC-CM on the inflammatory/resolution process through the IL-6 and IL-10 network [30].

The study of HSP expression by Saos-2 cells did not reveal any effects related to the presence of CM. It was interesting to study the expression of these proteins because a link seems to exist with periodontitis. Indeed, the expression of some HSP varies with the onset and evolution of periodontitis. We therefore examined the possible role of Saos-2 in this expression. HSP60, 70, and 90 can induce tissue pathology and play important role in many aspects of inflammation. They have been implicated in macrophage activation, which in turn results in *TNF* gene induction [66]. On the contrary, HSP27 has been seen to exert anti-inflammatory activities [27].

HSP70 is the main HSP expressed in the inflamed tissues [25]. They play a role in the induction of pro-inflammatory cytokines and may therefore contribute to the pathogenesis of chronic inflammatory diseases [67]. Their levels seem to vary according to the stages of evolution of the periodontitis, showing their implication in its onset and its progression. HSP70 can be considered as a potential marker for the severity of periodontal disease [25]. However, the mechanisms involved remain to be elucidated [25,26]. At the same time, this protein also seems to have a cell protective effect and might play a crucial role in the maintenance of periodontal tissue homeostasis and control of periodontal ligament physiology [68].

HSP60 has been proposed as a danger signal of stressed or damaged cells. Even if its role in the pathogenesis of periodontal disease is not fully elucidated, a pathological role should be expected, as its expression is abundant in the periodontal lesion and it stimulates the innate immune system [24,26,66].

HSP27 is a major intracellular molecular chaperone and controller of intracellular responses to inflammatory signals, preventing the release of inflammatory cytokines [27]. It seems to have a supportive role in the regeneration process related to cell migration [69]. Systemic concentrations may differ between different types of periodontal disease. Kaiser et al. showed that patients diagnosed with aggressive periodontitis had significantly lower levels of HSP27 in their circulation than patients with chronic periodontitis, suggesting that this protein may be regulated differently in the various forms of periodontitis [27].

To our knowledge, no study has established an association between HSP90 and periodontal disease. However, it is one of the most abundant HSP, expressed ubiquitously in a variety of cell types, including osteoblasts [70]. Its expression varies during osteoblastic differentiation [71].

## 5. Conclusions

The results of this study showed an enhancement of Saos-2 proliferation under the influence of hPDLSC-CM and an increase in some markers of osteoblastic differentiation, but this differentiation was not complete. However, Saos-2 cells were involved in the initial inflammation process by releasing IL-6 and activating COX2 in order to attract the debridement cells and the cells of repair. Regarding the variations in HSP expression observed in periodontitis, Saos-2 cells were not involved. The regenerative effect of MSC-CM seems to come from a cumulative effect of effective components, cytokines, and growth factors rather than a few factors present at high levels. Further in vitro, preclinical, and clinical studies are needed to improve the clinical efficacy of MSC-CM-based periodontal regeneration.

## Figures and Tables

**Table 1:** Description and characteristics of primers used for RT-qPCR. Abbreviations: ALP, alkaline phosphatase; COL1, collagen 1; OCN, osteocalcin; OP, osteopontin; RunX2, Runt-related transcription factor 2; COX2, cyclooxygenase-2; VEGF, vascular endothelial growth factor; HSP, heat shock protein; IL, interleukin, BSP, bone sialoprotein.

Amorce (Primer)	Sequence 5′-3′	Exon Position	Product Size (bp)	Primer Efficiency, *E*_p_ (%)	Coefficient of Determination, *R*^2^
*18S*	F: ATTAAGGGTGTGGGCCGAAG R: GGTGATCACACGTTCCACCT	F: E1/2 R: E2/3	111	96.1	1
*HPRT*	F: AGCTTGCTGGTGAAAAGG R: TCATTATAGTCAAGGGCATATC	F: E6/7 R: E8	107	97.6	0.99
*ALP*	F: AGAACCCCAAAGGCTTCTTC R: CTTGGCTTTTCCTTCATGGT	F: E7 R: E8	74	99.2	1
*COL1*	F: CGAAGACATCCCACCAATCAC R: TGTCGCAGACGCAGAT	F: E1/2 R: E2	98	99.7	0.999
*OCN*	F: GCAGCGAGGTAGTGAAGAGA R: GATGTGGTCAGCCAACTCGT	F: E3 R: E4	137	93.9	0.99
*OP*	F: TCACCTGTGCCATACCAGTTAAA R: GCCACAGCATCTGGGTATTTG	F: E2/3 R: E4	85	101.8	0.99
*RunX2*	F: ACCCAGAAGGCACAGACAGAAG R: AGGAATGCGCCCTAAATCACT	F: E5/6 R: E6	82	99.7	1
*COX2*	F: TGCGCCTTTTCAAGGATGGA R: CCCCACAGCAAACCGTAGAT	F: E6 R: E7	134	91.7	0.98
*VEGF*	F: TTGCCTTGCTGCTCTACCTCCA R: GATGGCAGTAGCTGCGCTGATA	F: E1 R: E3	126	96.9	1
*HSP27*	F: TGGATGTCAACCACTTCGCC R: ATGTAGCCATGCTCGTCCTG	F: E1 R: E2	106	106.6	0.93
*HSP60*	F: GACGACCTGTCTCGCCG R: ATCTGGCGAAAGACTGTGGG	F: E1 R: E2	78	106.5	1
*HSP70*	F: TTGTGCAGTTGCCTACAGGA R: GCAGTCACTTGCTCAGTGGT	F: E3 R: E4	85	103.6	1
*HSP90*	F: GATCACTTGGCAGTGAAGCATT R: GAGCACGTCGTGGGACAAAT	F: E6/7 R: E7	79	90.8	1
*IL-6*	F: CCAGAGCTGTGCAGATGAGTA R: TGGGTCAGGGGTGGTTATTG	F: E4 R: 35	89	95.2	1
*IL-8*	F: ACCACCGGAAGGAACCATCT R: AGCACTCCTTGGCAAAACTG	F: E1 R: E2	121	93.5	0.99
*BSP*	F: AACGAAGAAAGCGAAGCAGAA R: TCTGCCTCTGTGCTGTTGGT	F: E7 R: E7	77	96.2	0.99

**Table 2:** hPDLSC surface antigen expression (%) analyzed by flow cytometry.

	CD34	CD45	CD73	CD90	CD105
hPDLSC	0.36	0.34	99.97	99.97	96.77
Control	0.17	0.05	0.26	0.08	0.45

## Data Availability

The data presented in this study are available upon request from the authors.
